# A Novel Composite Voltammetric Sensor Based on Yttria-Stabilized Zirconia Doped with Neodymium-Carbon Black-Nafion Glassy Carbon Electrode for Metoprolol Determination

**DOI:** 10.3390/membranes13120890

**Published:** 2023-11-28

**Authors:** Małgorzata Suchanek, Beata Paczosa-Bator, Robert Piech

**Affiliations:** Department of Analytical Chemistry and Biochemistry, Faculty of Materials Science and Ceramics, AGH University of Krakow, Al. A. Mickiewicza, 30-059 Krakow, Poland; paczosa@agh.edu.pl

**Keywords:** metoprolol, yttria-stabilized zirconia doped with neodymium, voltammetry, flow injection analysis, pharmaceutical formulation

## Abstract

For the first time, a new composite voltammetric sensor based on yttria-stabilized zirconia doped with neodymium-carbon black-Nafion glassy carbon electrode (YSZNd-CB-Nafion/GCE) for the determination of metoprolol (MET) has been developed. The instrumental parameters and supporting electrolyte were optimized. For 105 s accumulation time, linearity was achieved in the range of 0.01 to 0.2 µM. The limit of detection (for 105 s accumulation time) was equal to 2.9 nM (2 µg/L), and was the best result in comparison to other voltametric sensors. The reproducibility of the metoprolol signal presented as relative standard deviation (RSD) was equal to 1.9% (*n* = 7). Additionally, our electrode is characterized by high stability, is easy to use, and has a short preparation time. The proposed sensor was found useful for MET determination in plasma and urine, as well as for pharmaceutical samples, with a good recovery parameter (96–108%). Flow injection analysis (FIA) with amperometric detection was also performed for MET determination. The recovery was calculated and was in the range 101–103%, suggesting that the proposed material may be applied in flow injection analysis.

## 1. Introduction

**Figure 1:**
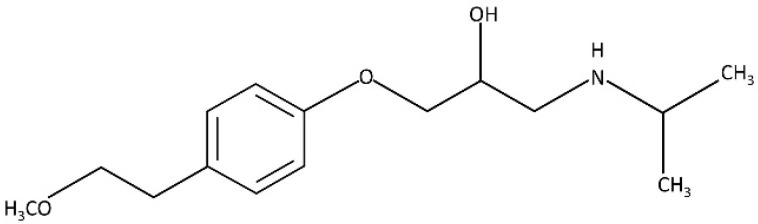
Chemical structure of metoprolol.

Nifedipine, nimodipine, ramipril, amlodipine and metoprolol are commonly used as antihypertensive and antianginal agents in patients at risk of stroke. All used as monotherapy caused statistically significant reductions in arterial pressure [1,2,3]. One of the most commonly used drugs, with a broad spectrum of action, is metoprolol. Metoprolol, chemically known as (1-isopropylamino-3-[4-(2-methoxyethyl)phenoxy]propan-2-ol) (Figure 1), is one of the selective beta-blockers, and its action involves blocking beta receptors, mainly β1. It is used in the treatment of various cardiovascular conditions such as hypertension, angina pectoris and cardiac arrhythmias, as well as myocardial infarction. The International Olympic Committee has added this drug to the list of doping agents, due to its sedative effect [4]. Metoprolol (MET) is an important drug for improving the survival of a person after a heart attack, but an overdose of such a β-blocker can lead to bradycardia, bronchospasm, hypoglycemia, hypotension, and also provocation of cardiac failure. Compared to propranolol, metoprolol has a weak effect on inhibiting isoprenaline-induced tachycardia, and it reduces heart rate during exercise to a greater extent than β-blockers with intrinsic sympathomimetic activity. In addition, cardiovascular diseases were the most common cause of death among metoprolol users, particularly in older people above 50 years old [5]. Therefore, it is important to develop simple, sensitive, cost-effective, and selective analytical methods with low detection limits suited to human biofluids and medicinal products.

The determination of metoprolol has been described in the literature by different methods, including absorption spectroscopy [6], spectrophotometry [7,8], high-performance liquid chromatography with UV detection (HPLC-UV), fluorescence and mass spectrometry [9,10,11], capillary electrophoresis [12], and fluorimetry [13,14]. Voltammetry can also be employed for MET determination [15,16,17]. Voltammetry is a method characterized by a high sensitivity, the ability to achieve low detection limits and low consumption of analyte as well as by cost-effective analysis. All electrochemical processes occur on the working electrode. In voltammetry, many types of working electrodes can be applied for the inorganic as well as organic substance determination [18,19,20,21]. A very popular electrode use in voltammetry is the glassy carbon electrode (GCE) with various surface modifiers. For metoprolol determination, different types of electrodes were used, e.g., a hanging mercury drop electrode (HMDE), glassy carbon electrode (GCE) [22], carbon paste electrode, boron-doped diamond electrode (BDDE) [17], and graphite electrode (GRE) [23]. In recent years, scientists have focused on modifying solid electrodes in order to improve their performance. Modification of the surface makes it possible to increase a specific surface area, improving electrical conductivity and facilitating electron transfer. Modifications of the electrode surfaces can be performed in various ways, e.g., by using carbon nanotubes [24,25,26], conducting polymers [16,27], metal nanoparticles [28,29] or yttria-stabilized zirconia (YSZ) [30,31].

Carbon black (CB) is attracting interest all over the world, due to its properties and application (a black pigment in coating materials, it reinforces rubber and plastic mixtures). Carbon black is formed as a result of incomplete combustion of petroleum products. This powder, consisting of carbon, is characterized by a high reversible capacity, large surface area, low density and disordered structure. Using carbon black as a modifier layer of electrodes has achieved lower detection limits, and it provides stable signals with high precision and repeatability of measurements [32]. Carbon black in voltammetry is applied to estradiol [33], melatonin [34] or solid residues of lead [35] determination.

Nafion, a perfluorinated sulfonated cation exchanger, consists of a linear backbone of fluorocarbon chains and ethyl ether side groups with sulfonated cation exchange sites. It is used as a modifying medium in sensor production, due to its antifouling ability, chemical inertness, thermal stability, mechanical strength and high permeability [36,37]. The advantage of carbon black comminated with Nafion as a modifying layer is to achieve a large working surface area, consequently lowering the detection limits of voltametric sensors compared to those based on other modification materials [19,38,39,40].

Pure zirconia is crystallized in monoclinic and tetragonal, as well as cubic forms, depending on the temperature. Through the addition of various oxides, the high-temperature structure of zirconium oxide polymorphs can be synthesized at room temperature. Yttrium oxide is one of the components introduced into the zirconia structure. The zirconia stabilized with yttria can be applied as an electrolyte in the SOFC (solid oxide fuel cells). The tetragonal and monoclinic structure in synthesis at room temperature can be observed through the addition of another percentage of yttria to the structure [41]. The yttrium oxide-doped zirconium oxide solid solution (YSZ) can be modified by rare earth oxides, such as lanthanum, cerium, ytterbium, samarium, gadolinium or neodymium [42,43,44,45]. Modification of the structure of YSZ with Nd causes improvement in mechanical and optical properties [46]. The yttria-stabilized zirconia doped with neodymium (YSZNd) was used in adsorption processes for humic acid removal from an aqueous solution [47]. For the first time, YSZ doped with Nd has been used for modification of a glassy carbon electrode.

The main aim of this work is to present for the first time the analytical application of YSZNd-CB-Nafion composite modified glassy carbon electrode in the determination of metoprolol in different kinds of samples. A combination of carbon nanomaterial, YSZNd nanopowder and polymer-Nafion increases the working surface electrode, but it also ensures approximately 15 min for working electrode preparation time for the voltametric measurement, due to its accelerated drying process. Additionally, our solution presents the best results for LOD value in comparison to other voltammetric methods for metoprolol determination. The usefulness of the proposed sensor was confirmed through metoprolol determination in the pharmaceutical product and urine and plasma samples. Moreover, the presented electrode can be used in flow injection measurements in order to analyze samples in a very short period of time.

## 2. Materials and Methods

### 2.1. Apparatus

For all voltametric measurements, a specialized multipurpose Electrochemical Analyzer type M161 connected to an electrode stand type M164 (mtm-anko, Krakow, Poland) with dedicated EAQt software (Krakow, Poland) was utilized. The standard voltammetric system cell with a volume of 20 mL, a double-junction Ag/AgCl/KCl (3 M) as reference electrode, the platinum spiral rod as the auxiliary electrode, and the glassy carbon electrode (GCE) diameter of 3 mm (Mineral, Łomianki-Sadowa, Poland) or modified with spherical carbon black nanoparticles, Nafion and yttria-stabilized zirconia doped with neodymium (YSZNd-CB-Nafion/GCE) as a working electrode was applied. Voltammograms were visualized and then performed with Matlab R2019a. The electrolyte in the cell was stirred between measurements using a Teflon-coated stir bar with a metal core and a magnetic stirrer. The Elmetron CX-705 multi-function meter (Elmetron, Zabrze, Poland) was used for pH measurements of the buffer solution. An ultrasonic bath was used for sonification of the solvents (Intersonic, IS-1K, Olsztyn, Poland).

The electrical impedance spectroscopy measurements (EIS) were performed using the VersaSTAT4 (Ametek, Berwyn, PA, USA). The three-electrode system with a double-junction Ag/AgCl/KCl (3 M) as a reference electrode, a glassy carbon rod as the auxiliary electrode, and GC electrodes modified with carbon black, Nafion, Nafion-Carbon Black and YSZNd-Carbon Black-Nafion as a working electrode were used for the measurements. All data analyses were performed with ZSimpWin 3.60 (Echem Software, Ann Arbor, MI, USA) software.

The flow system was composed of a 0.05 L solution reservoir, and an 800 Dosino pump connected to 900 Touch control panel (Metrohm, Herisau, Switzerland), as well as a sample injection valve (Rheodyne Model 7010) and also a flow wall-jet detector. A thin-layer amperometric detector with a glassy carbon disc electrode 1 mm in diameter was used as well.

### 2.2. Chemicals and Glassware

All used chemicals were of analytical or HPLC grade and were used as received, without further purification. The standard solution of MET (0.01 M) was obtained from certified reference material purchased from Sigma Aldrich (St. Louis, MO, USA) by dissolution in double-distilled water, and was protected from the light and stored in a refrigerator between measurements. The MET solution was stabilized by the addition of acetic acid (Merck KGaA, Darmstadt, Germany). Solutions with 0.001 M and 0.0001 M of MET concentrations were prepared daily by dissolving an appropriate amount of reagent in double-distilled water. The other reagents were purchased as follows: Carbon black CAT from 3D-nano (Poland), ethanol (POCH, Gliwice, Poland), and proton-exchange polymer Nafion—5% solution (Sigma Aldrich, St. Louis, MO, USA). Zirconium oxychloride octahydrate (ZrOCl·8 H_2_O) as a powder, yttrium oxide (Y_2_O_3_), and neodymium oxide were obtained from Sigma Aldrich and utilized for nanopowder preparation of 3 mol % yttria-stabilized zirconia doped with neodymium (YSZNd). Freeze-dried urine was obtained from Medichem (Steinenbronn, Germany), while human plasma was obtained from Biowest (Naualle, France). Citric acid, lactose monohydrate, aspartame, starch, magnesium stearate, talc, cellulose, titanium dioxide, glucose, caffeine, ascorbic acid, uric acid, acetaminophen (Merck, Darmstadt, Germany), and Triton X-100 (Windsor Laboratories Ltd., UK (Kingston, Jamaica) were used for interference studies. All solutions were dissolved in double-distilled water.

### 2.3. Synthesis of YSZNd Nanopowders

The procedure of synthesis of 3 mol. % yttria-stabilized zirconia doped with neodymium (YSZNd) nanopowders was prepared using the hydrothermal method described in previously published work [47]. Zirconium oxychloride octahydrate, yttrium oxide and neodymium oxide were mixed in nitric acid to obtain the ZrO_2_:Y_2_O_3_:Nd_2_O_3_ ratio 97:2:1 mol %. The received solutions were precipitated in the presence of ammonia water solution. After washing the obtained gel with double-distilled water to eliminate ammonium chloride and nitrate salts, the co-precipitated gel underwent hydrothermal treatment in water vapor pressure (4 h, 250 °C) and was then dried at 50 °C for another 4 h.

### 2.4. Preparation of the Modified Electrodes

The modifier suspension was obtained by weighing 5 mg of spherical carbon black and 5 mg of YSZNd nanopowder, which was quantitatively transferred into a 5 mL volumetric flask. The optimized amount of YSZNd nanopowder was sonicated for about 15 min. in a suspension of 100 µL of Nafion (5% solution) and ethanol (96%).

In the second step, the GCE was modified with YSZNd-CB-Nafion fabrication. The surface of the GCE was burnished on a 0.3 µm Al_2_O_3_ slurry and then thoroughly rinsed with a double-distilled water stream. Next, it was immersed in the methanol-water solution and subjected to sonication for 3 min. After drying, the GCE electrode surface was modified by applying 5 µL of the previously prepared suspension. After 15 min of drying at room temperature, the prepared electrode was ready to use for two weeks.

### 2.5. Sample Preparation

#### 2.5.1. Pharmaceutical Samples

Pharmaceutical products (containing 50 mg metoprolol tartrate per tablet) were purchased from the local pharmacy. For measurement, three tablets were crushed in an agate mortar and then quantitatively transferred to a volumetric flask (20 mL) and dissolved in double-distilled water. After complete dissolution and homogenization, the tablet suspension was filtered through syringe filters with pore size 0.45 µm purchased from Biosens (Warsaw, Poland).

#### 2.5.2. Urine

A freeze-dried urine reagent for commercial use was purchased from Medidrug (Barcelona, Spain) and prepared according to the instructions obtained by the manufacturer. The vial’s content was diluted with double-distilled water and passed through an RC syringe filter (pore diameter 0.45µm) and then used for metoprolol determination [18,19,48].

#### 2.5.3. Plasma

A plasma sample for commercial use was purchased from Biowest and protected from the light and stored in a freezer at −20 °C. For removal of interfering proteins from the samples, the plasma was prepared using the following procedure: 800 µL of plasma was blended with 200 µL of trichloroacetic acid (TCA—10%) and vibrated for 2 min on the vortex. The suspension was separated by means of centrifugation at 10,000 rpm, and was then filtered through syringe filters (pore size 0.45 µm) and used in measurements [18,48,49].

### 2.6. Measurement Procedure

For quantitative measurements of metoprolol, the differential pulse voltammetry (DPV) technique was used. The appropriate quantities of MET standard solution were introduced into a 20 mL voltammetric cell, and the acetate buffer was diluted up to 10 mL (0.1 M concentration), with pH 4.0, and the measurement was carried out. A magnetic stirrer with an approx. speed of 500 rpm was used to accumulate MET onto the electrode surface during the preconcentration step. To characterize the properties of the proposed YSZNd-CB-Nafion/GCE, a cyclic technique was applied. The instrumental parameters for the DPV were also optimized, and were as follows: sampling and waiting time t_p_ = t_w_ = 20 ms, step potential E_s_ = 5 mV, pulse amplitude height dE = 35 mV. The voltammograms were recorded by scanning the potential in the range of 900 to 1400 mV. The preconcentration step was described using the parameters E_acc_ = 900 mV and t_acc_ = 45 s. In order to achieve a high repeatability of the MET signal, a rest period of 15 s between each voltammogram registration was mandatory.

## 3. Results

### 3.1. Sensor Characterization

**Figure 2:**
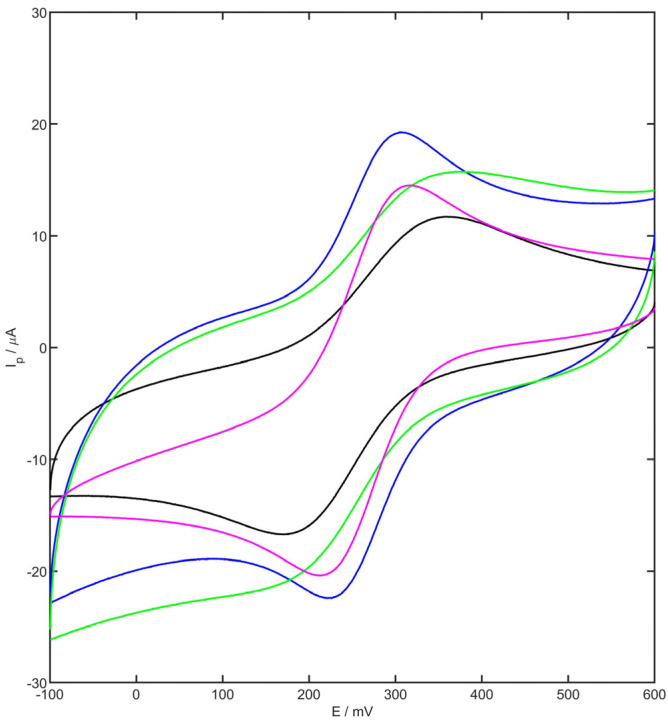
Cyclic voltammogram of 1 mM K_3_[Fe(CN)_6_] in 1 M KCl for GC electrode (black), CB/GC electrode (blue), CB-Nafion/GC electrode (green), and YSZNd-CB-Nafion/GC electrode (purple).

The electrochemical behavior of the YSZNd-CB-Nafion modifier layer on the surface of the GC electrode was investigated. Cyclic voltammograms (CVs) of the bare glassy carbon electrode, GC modified with CB, GC modified with CB and Nafion, and GC modified with CB, Nafion and YSZNd were registered in 1mM of K_3_[Fe(CN)_6_] in 1 M KCl. The registered voltammograms for each presented electrode with a scan rate 100 mV/s are shown in Figure 2. Based on the Randles–Ševčik equation (Equation (1)), the electrochemically active surface area of each tested electrode was computed [50]:

(1) $$I_{p}=\left(2.69\times 10^{5}\right)n^{3/2}A_{el}D^{1/2}v^{1/2}c$$

where *I_p_* is a peak current, *A_el_* is the electrochemically active surface area of the electrode and *v* is the scan rate (v=100 mV/s), $D=7.6\times 10^{-6}$ cm^2^/s.

The voltammogram with symmetric anodic and cathodic peaks (8.81 µA and 9.75 µA, respectively) was recorded at the bare GCE. The modification of the electrode with carbon black caused the anodic and cathodic current to increase to 12.11 µA and 11.44 v µA, respectively, compared with the unmodified electrode. For the carbon black-Nafion layer, the observed peaks were widened, and the values of the redox peak potential and the current were not obvious. The YSZNd-carbon black-Nafion/GCE showed an increase in the anodic and cathodic peak current values (15.15 µA and 14.61 µA, respectively). The computed electrochemically active surface area of YSZNd-CB-Nafion/GCE (0.065 cm^2^) was ca. 25% higher than the CB/GCE (0.052 cm^2^), and twice as large as for the CB-Nafion/GCE (0.038 cm^2^) and bare GCE (0.032 cm^2^). The significant increase in the electrochemically active surface area of YSZNd-CB-Nafion/GCE, accompanied by facilitated electron transfer, allows for an increase in the efficiency of the occurring electrode reaction, resulting in an increase in the registered current and, consequently, in higher sensitivity.

**Figure 3:**
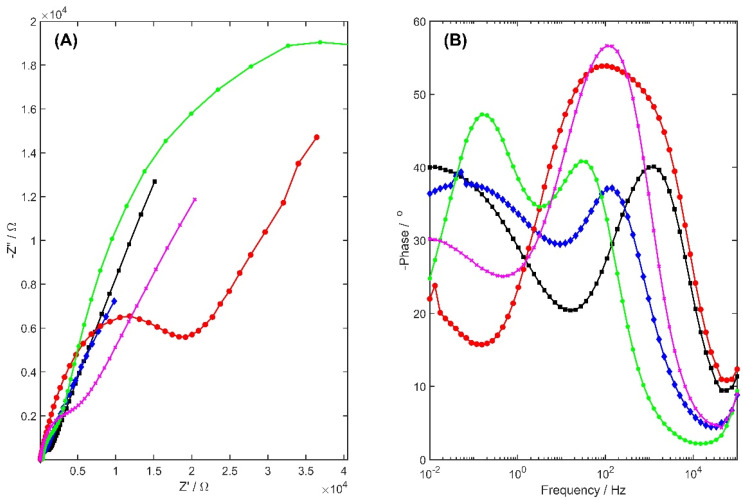
(**A**) Nyquist and (**B**) Bode plots recorded for 1 mM K_3_[Fe(CN)_6_] in 0.1 M KCl solution for GC electrode (black), CB/GC electrode (blue), Nafion/GC electrode (red), CB-Nafion/GC electrode (green), and YSZNd-CB-Nafin/GC electrode (purple).

The electrical impedance spectroscopy measurements (EIS) were performed for 1 mM K_3_[Fe(CN)_6_] in 0.1 M KCl, using the sinusoidal signals of frequency ranging from 100 kHz to 10 mHz and the small excitation amplitude of 10 mV peak to peak, applied to the formal potential of K_3_[Fe(CN)_6_]. The obtained Nyquist plots for the unmodified glassy carbon electrode and for those modified with carbon black, Nafion, carbon black-Nafion, and yttria-stabilized zirconia doped with neodymium-carbon black-Nafion are presented in Figure 3. The charge transfer resistance (R_ct_), and capacitance (*C_eff_*) were determined according to the obtained EIS spectra. The capacitance was computed using data from the ZSimpWin 3.60 and the following equation:

(2) $$C_{eff}=Y_{0}^{1/N}\times {\left[\frac{1}{R_{S}}+\frac{1}{R_{P}}\right]}^{\frac{N-1}{N}}$$

The calculated parameters were presented in Table 1. 

The recorded EIS spectra show that the modification of the GCE surface with Nafion contributes to a distinct increase in R_ct_ and a slight increase in capacitance, C. Modification of the GCE surface with YSZNd-Nafion-CB caused a significant increase in capacitance, and the value of the charge transfer resistance was highly decreased, compared to the GC electrode with Nafion, which favors a higher electrode-reaction efficiency.

Moreover, a significant change in the distance of the low- and high-frequency arcs for the reaction on the bare GC electrode and the GC electrode with modifiers was observed. Two characteristic frequencies were observed on the Bode plots at low and high values, which indicated a clear separation of electrode processes in both frequencies (Figure 3B). For the GC electrode, two dominant electrode processes were observed, associated with the low-frequency arc ~10 mHz and with the high-frequency arc ~1300 Hz. Modification of the carbon black decreased the high-frequency arc slightly to ~140 Hz, and increased the low-frequency arc to ~70 mHz. The addition of Nafion to carbon black as the modifier of the GC electrode caused an increase in the low-frequency arc to 140 mHz, and a decrease in the high-frequency arc to 30 Hz. The characteristic frequency of the electrode process at the low-frequency arc was 10 mHz at YSZNd-CB-Nafion/GCE, and this increased to 140 Hz at the high-frequency arc. For the Nafion-modified GC electrode, there was only one dominant electrode process associated with the high-frequency arc, and its characteristic frequency was ~100 Hz.

### 3.2. Effect of Modifier

**Figure 4:**
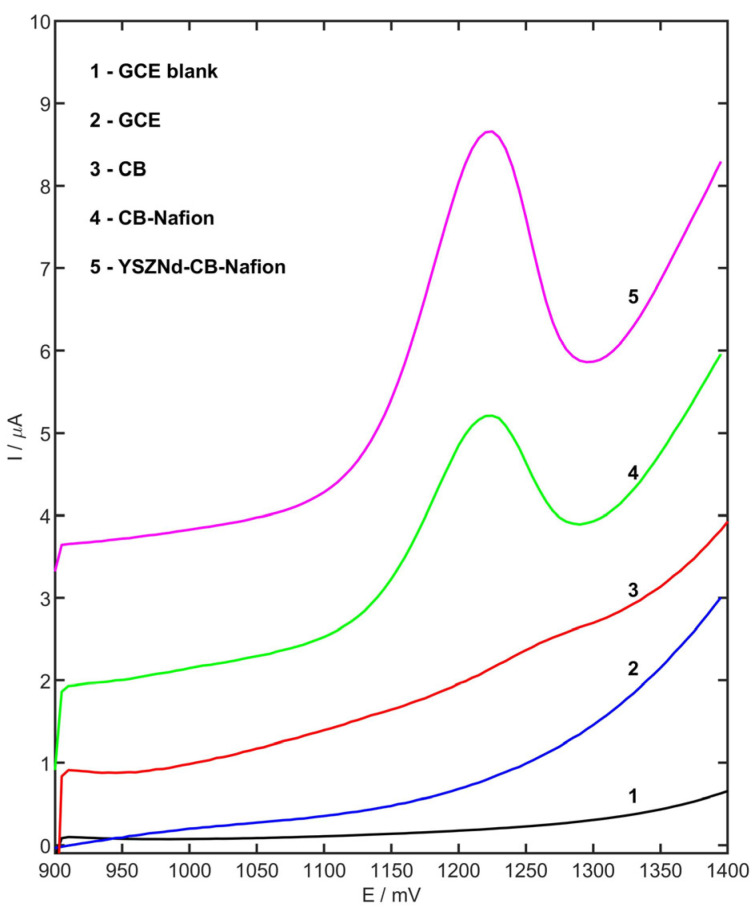
Stripping differential pulse voltammograms in 0.1 M acetate buffer (pH 4.0) onto GCE (1), and 2.5 µM MET in 0.1 M acetate buffer (pH 4.0) onto GCE (2), CB/GCE (3), CB-Nafion/GCE (4), YSZNd-CB-Nafion/GCE (5). Preconcentration potential and time were equal to 900 mV and 45 s, respectively.

The electrochemical oxidation of MET was examined using stripping differential pulse voltammetry with the accumulation step (E_acc_ = 900 mV, t_acc_ = 45 s) in the acetate buffer (pH 4.0) on the surface of the GCE, CB/GCE, CB-Nafion/GCE, and YSZNd-CB-Nafion/GCE. A well-defined oxidation peak of MET was observed on electrodes modified by carbon black and Nafion and YSZNd. Figure 4 clearly shows that the peak potential was lower for YSZNd-CB-Nafion/GCE compared to CB-Nafion/GCE (1215 mV and 1225 mV, respectively). This may indicate that the electrodes modified by YSZNd have catalytic properties. The peak current recorded on the YSZNd-CB-Nafion/GCE, CB-Nafion/GCE, CB/GCE, and bare GC electrodes were equal to 4.15, 1.38, 0.28, and 0.02 µA, respectively.

**Figure 5:**
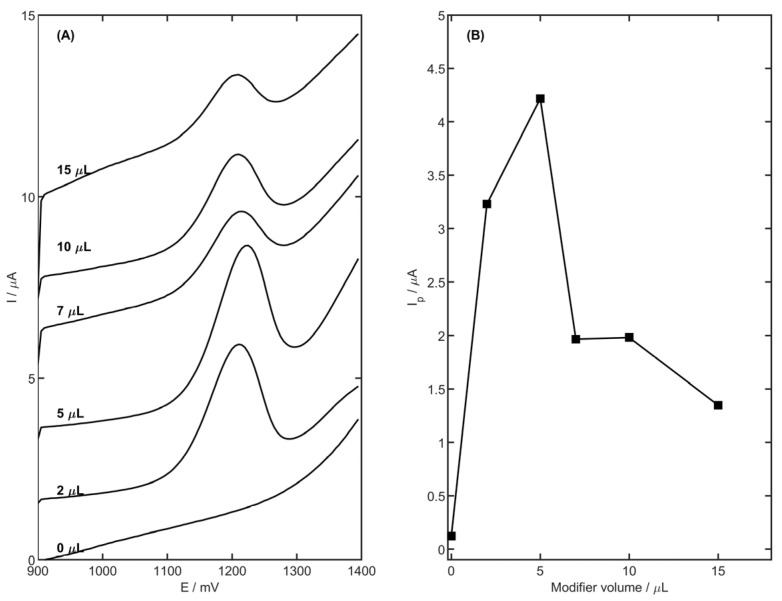
The dependence of MET peak current on volume of YSZNd-CB-Nafion modifier on the surface of GCE, DP voltammograms (**A**), plot (**B**). The electrolyte was of 0.1 M acetate buffer with pH = 4.0, and the MET concentration was equal to 2.5 µM, E_acc_ = 900 mV, t_acc_ = 45 s.

In order to obtain the highest sensitivity and maintain stability of the registered signal, optimization of the volume of the surface modifier was applied. Six GCEs were prepared and modified with the following volumes of YSZNd-CB-Nafion suspension: 0, 2, 5, 7, 10 and 15 µL. The signal was measured for 2.5 µM MET (in 0.1 M acetate buffer—pH 4.0). The results presented in Figure 5 show that the maximum MET peak current was observed for 5 µL of suspension. For a smaller volume (in the range 2–5 µL) of the modifier, a high peak current of MET was observed. The larger volumes of suspension caused a visible decrease in the peak current of MET, and was equal to 1.35 µA for 15 µL of volume of suspension. The decreased MET peak current for the larger volumes of suspension could be caused by the suspension blocking of the electrode surface. Moreover, the higher capacitance current was observed for larger volumes of suspension. Therefore, this value (5 µL) was chosen as optimal for subsequent measurements.

### 3.3. Influence of the Composition of the Supporting Electrolyte on MET Signal

**Figure 6:**
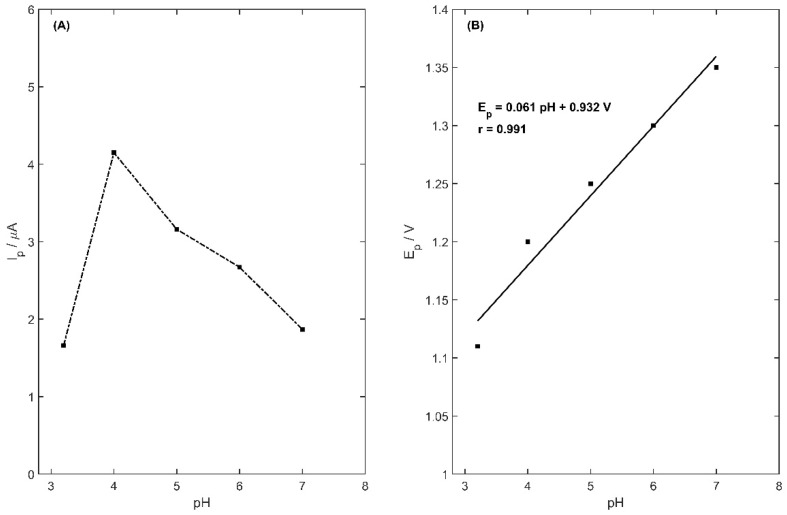
Metoprolol current peak (**A**) and potential peak (**B**) dependence on supporting electrolyte pH in the range 3.2–7.0.

The type of supporting electrolyte is an important element for metoprolol determination using the voltammetry method. It has an influence on the value of the peak current and also the peak potential. The optimization of the supporting electrolyte was conducted for 2.5 µM of MET concentration and accumulation potential and time were equal to 900 mV and 45 s, respectively. Five types of supporting electrolytes were examined: acetate buffer (0.1 M pH 4.0), phosphate buffer (0.1 M pH 5.0), KCl (0.1 M pH 5.0), ammonia buffer (0.1 M pH 8.2) and borate buffer (0.1 M pH 9.1). In the case of borate buffers, no signal of MET was registered. The MET peak was recorded in the electrolytes: acetate buffer: I_p_ = 4.15 ± 0.10 µA, E_p_ = 1215 mV; phosphate buffer: I_p_ = 1.14 ± 0.14 µA, E_p_ = 1340 mV; KCl: I_p_ = 1.04 ± 0.05 µA, E_p_ = 1351 mV; and ammonia buffer: I_p_ = 1.51 ± 0.18 µA, E_p_ = 1361 mV. The highest current peak was registered for the 0.1 M acetate buffer. The changes in the MET signal were measured, and also depended on the pH of the acetate buffer. The dependence of the peak current on the supporting electrolyte pH in the range 3.2–7 is presented in Figure 6A. The highest peak current was registered for pH 4.0 of the acetate buffer, and therefore it was chosen for further measurements.

### 3.4. Electrochemical Behavior of MET on YSZNd-CB-Nafion GCE Electrode

**Figure 7:**
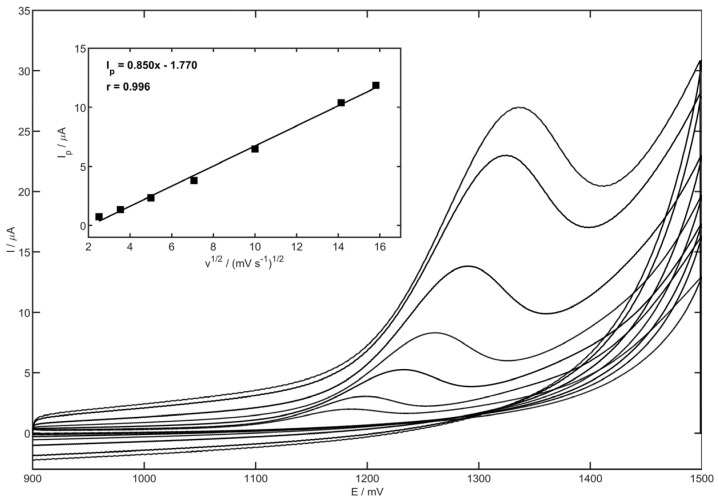
Cyclic voltammograms of 10 µM MET in 0.1 M acetate buffer (pH 4.0) measured on the glassy carbon electrode modified with YSZNd, carbon black and Nafion. Scan rate value: 6.3, 12.5, 25, 50, 100, 200 and 250 mV s^−1^.

Measurements were carried out in the acetate buffer as the supporting electrolyte containing 10 µM of MET. Cyclic voltammograms were registered by changing the scan rate in the range of 6.3 to 250 mV s^−1^ (potential range of 900 to 1500 mV). The results demonstrated (Figure 7) no reduction peak in the recorded cathodic scan, which suggests that the metoprolol oxidation process on the YSZNd-CB-Nafion/GCE electrode is irreversible. The dependence of the MET peak current on the scan rate, as well as the square root of the scan rate, was plotted. The linear correlation was observed from the peak current on the square root of the scan rate plot, which suggests that metoprolol oxidation is caused by the diffusion-controlled process.

The number of electrons exchanged during the metoprolol oxidation reaction can be calculated using the following equation [51]:

(3) $$\alpha n=\frac{0.048}{\left|E_{p}-E_{p1/2}\right|}$$

The αn value computed from the above equation was equal to 0.96, assuming α as 0.5 and the number of electrons exchanged during the oxidation reaction to be ca. 2.

In addition, the dependence of peak potential on the pH of the supporting electrolyte was examined. In the experiment, five electrolytes of 0.1 M acetate buffer with pH in the range of 3.2 to 7 were tested. The metoprolol concentration was equal to 2.5 µM, with accumulation potential and time equal to 900 mV and 45 s, respectively. In the tested range of pH value, the linear dependence between the value of pH and the MET peak potential was observed (Figure 6B). The increase in pH value resulted in the shift in the peak of metoprolol towards more positive values, which can be described using the following equation:

(4) $$E_{p}=0.061\;pH+0.932\;V$$

**Figure 8:**
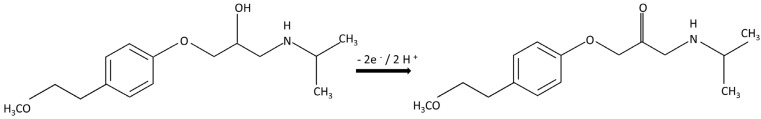
Proposed mechanism for electro-oxidation of MET on YSZNd-CB-Nafion/GCE.

The slope of the curve, the value of which was close to the theoretical 0.059 V pH^−1^, was observed. It proves that an equal number of exchanged protons and electrons are present in the MET oxidation process on the surface of the YSZNd-CB-Nafion/GC electrode. At this point, we could propose that the mechanism of metoprolol oxidation occurs at the hydroxyl group. The proposed mechanism of possible MET oxidation on the YSZNd-CB-Nafion/GC electrode is presented in Figure 8. Similar results have been reported in the electro-oxidations of MET [28], another electro-oxidation of propranolol [19,52], where the electro-oxidation occurred on the hydroxyl group with the transfer of two protons and two electrons.

### 3.5. Optimization of DPV Technique Parameters on MET Signal

The optimization of the DPV technique parameters is an important part of planning research, due to its impact on the sensitivity of the voltammetry signal. The measurements were carried out in the supporting electrolyte consisting of a 0.1 M acetate buffer (pH 4.0) and also 2.5 µM of the MET concentration. Between each recorded voltammogram, a rest period of 15 s was absolutely required in order to receive the high repeatability of the MET signals. The following parameters were investigated for a wide range of values: sampling time t_s_ (10–50 ms), waiting time t_w_ (10–50 mV), step potential E_s_ (1–10 mV), and pulse amplitude ΔE (5–100 mV—positive and negative mode). The best results were registered for the following values: t_s_ = t_w_ = 20 ms, E_s_ = 5 mV, and ΔE = 35 mV. These parameters were used in subsequent measurements.

### 3.6. Influence of Preconcentration Potential and Time on MET Signal

The preconcentration potential and time have significant impact on the stripping voltammetry analysis. Optimization of these parameters allows the low analyte concentration to be determined, and improves the sensitivity of the method. The experiment was carried out using a 0.1 M acetate buffer (pH 4.0) and 2.5 µM of MET. The preconcentration potential varied from −100 to 1100 mV. The experiment showed that the MET current does not depend on preconcentration potential. The preconcentration potential equal to 900 mV was selected for the determination of the MET.

**Figure 9:**
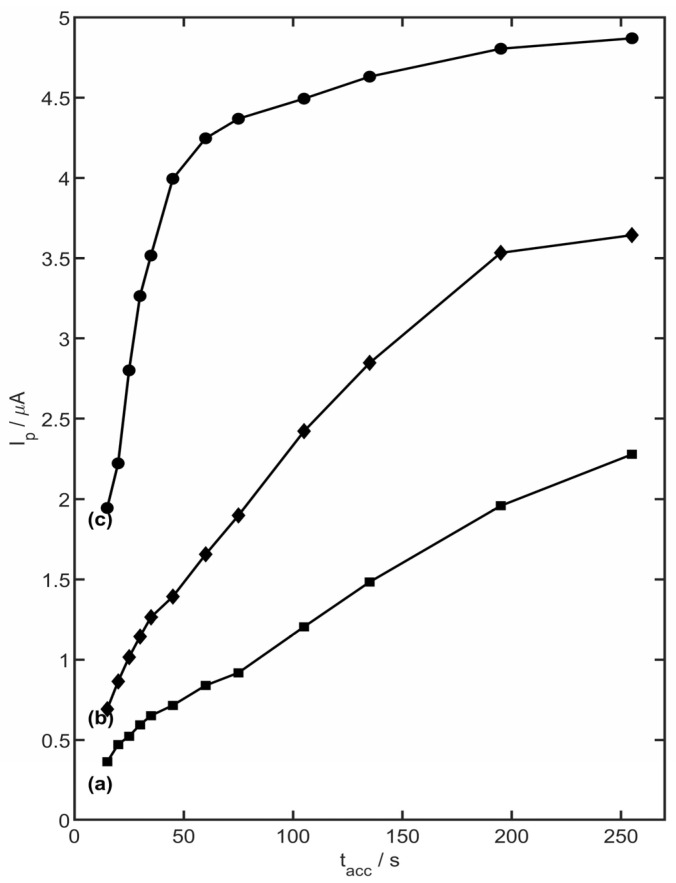
The dependence of MET peak current on accumulation time in the range of 15 s to 255 s for (a) 0.5 µM, (b) 1 µM, and (c) 2.5 µM metoprolol concentration in 0.1 M acetate buffer (pH 4.0).

The accumulation time was tested under the same conditions as the preconcentration potential for the 2.5 µM of metoprolol. Between each recorded voltammogram, a rest period of 15 s was absolutely required. The time was covered in the range of 15 to 255 s. The plot of the dependency between the metoprolol current peak value and accumulation time (t_acc_) value is presented in Figure 9. For all presented MET concentration values, increasing the value of the preconcentration time caused an increased value in the peak current. Moreover, the accumulation time was dependent on the concentration of metoprolol. The maximum obtained peak current for the MET concentration of 2.5 µM was equal to 4.24 µA ((t_acc_ = 60 s), while for 1 µM it was equal to 3.53 µA (t_acc_ = 195 s). For 0.5 µM of MET, the maximum peak current was observed for the accumulation time of 255 s, and was equal to 2.28 µA. A longer accumulation time was not tested, due to extended analysis time. The voltammetric response of the electrode was directly proportional to the surface concentration of the accumulated species. For the analytical performance studies, the time of 45 s was chosen to accumulate the metoprolol on the YSZNd-CB-Nafion/GC electrode.

### 3.7. Interferences

The influence of foreign ions on the metoprolol signal is a significant part of developing a new analytical method. In the research, the influence of the mentioned ions was investigated: Mg(II), Ca(II), K(I) (50 µM added), Cu(II), Pb(II), Zn(II), Mn(II) (5 µM added), SO_4_^2−^, NO^3−^, Cl^−^, and PO_4_^3−^ (1 mM added). The studies showed that the addition of cations and anions did not influence a difference in the MET peak current and potential. Moreover, organic compounds, as well as potential ingredients of the tablet, urine and plasma, were tested, such as: glucose, saccharose (50 µM added), citric acid (100 µM added), lactose monohydrate, ascorbic acid, uric acid, aspartame, caffeine, acetaminophen, starch, talc (concentration 20 µM each), magnesium stearate, microcrystalline cellulose, Titanium dioxide (5 mg per 10 mL of electrolyte), and Triton X-100 (2.5 ppm added). Only the addition of ascorbic acid, acetaminophen and citric acid caused a decrease in the peak current (16%, 9%, and 9%, respectively).

### 3.8. Analytical Performance

**Figure 10:**
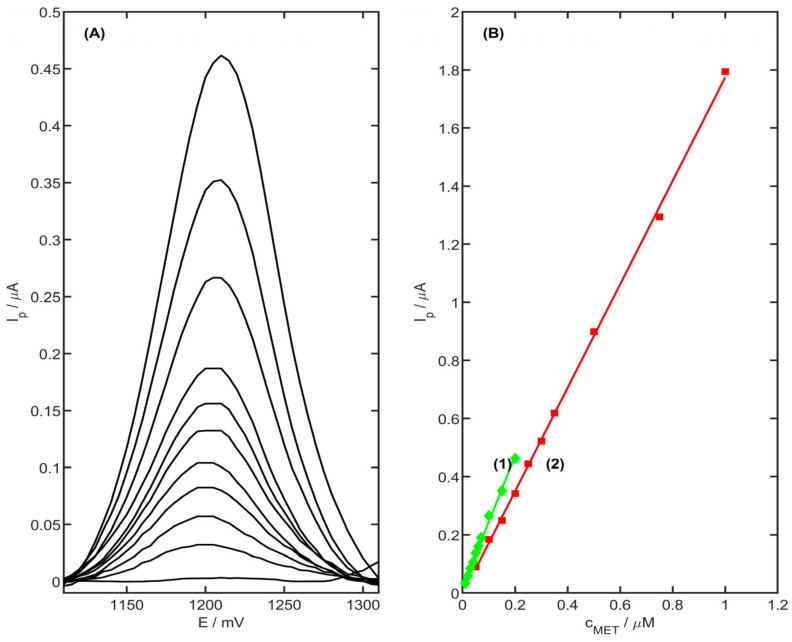
(**A**) DPV calibration voltammograms obtained for metoprolol concentrations in the range of 0.01 to 0.2 µM and the blank in 0.1 M acetate buffer (pH 4.0), preconcentration time 105 s. (**B**) Calibration curve registered for the concentration in the range (1) 0.01–0.2 µM (t_acc_ = 105 s) and (2) 0.05–1.0 µM (t_acc_ = 75 s).

Calibration is one of the parameters for investigating the analytical efficiency of the method. The obtained calibration voltammograms and also the calibration curves are shown in Figure 10. The experiment was carried out in a 0.1 M acetate buffer (pH 4.0) with the accumulation potential equal to 900 mV. The linearity was obtained for the metoprolol concentration in the range of 0.05–1 µM (Figure 10B) (r = 0.999; slope 1.78 ± 0.01 µA µM^−1^; intercept 0.02 ± 0.02 µA), and the detection limit (computed according to equation LOD = 3.3 SD/b, where SD is the standard deviation of current for a blank, and b is the slope of the calibration curve) was equal to 10.2 nM for the accumulation time equal to 75 s. The lowest detection limit was obtained for the metoprolol concentration in the range 0.01–0.2 µM and 105 s preconcentration time, and it was equal to 2.9 nM (slope of the regression line 2.25 ± 0.06 µA µM^−1^, intercept 0.02 ± 0.01 µM, r = 0.997), which is a very good result compared to other electrochemical sensors (Table 2). The limit of quantification (LOQ = 10 SD/b) was also computed, and was equal to 6.6 nM. The electrode reproducibility was also tested. The electrode was tested for 2.5 µM and 0.05 µM of MET concentration. The results show that the reproducibility of the electrode, presented as relative standard deviation (RSD), was equal to 1.9% and 1.15% (*n* = 7), respectively. The stable signal was observed for 60 measurements on one electrode.

**Figure 11:**
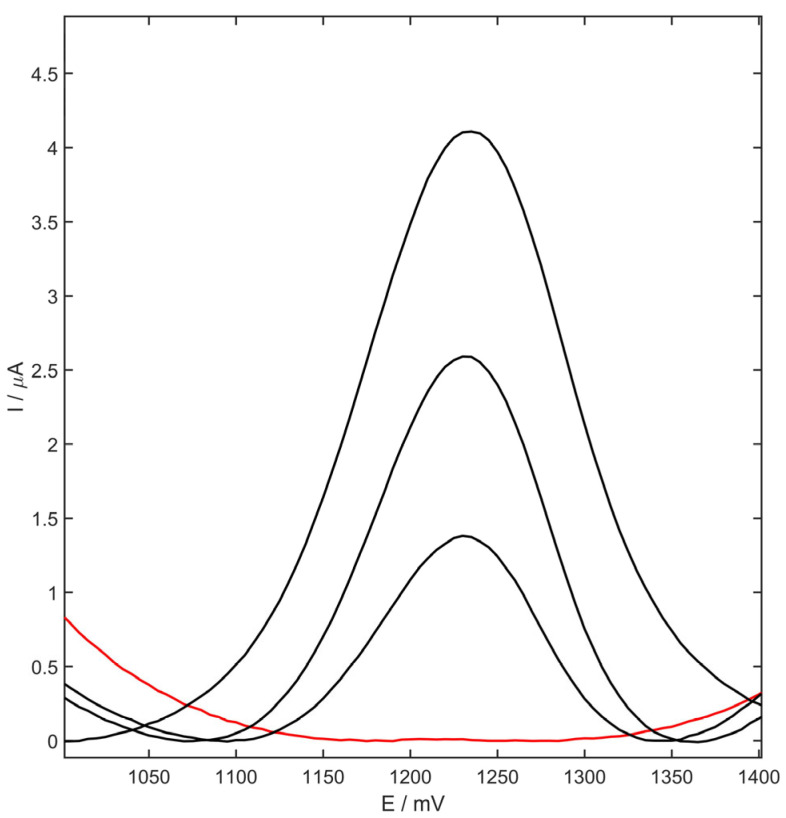
Voltammograms of metoprolol determination in urine sample (urine curve marked as red).

Furthermore, the application of the presented electrode and the procedure for metoprolol determination in the pharmaceutical sample, for urine as well as plasma, were tested. For this purpose, one pharmaceutical formulation containing MET was chosen. All measurements were conducted using the standard addition method and the procedure described in Section 2.6. The procedure of preparing the samples was described in Section 2.4. The results are presented in Table 3. The mass of metoprolol per tablet was calculated, and was equal to 49 ± 2 mg. The recoveries were in the range 96–108%, suggesting the analytical usefulness of yttria-stabilized zirconia doped with neodymium-carbon black-Nafion-modified GC electrode for metoprolol determination in various samples. The obtained voltammogram for the urine sample, along with standard additions of MET, is presented in Figure 11. The MET peak obtained in the presented sample of urine using glassy carbon electrode modified by YSZNd-Carbon Black-Nafion was clearly distinguished from the background.

### 3.9. Flow Injection Analysis with Amperometric Detection

**Figure 12:**
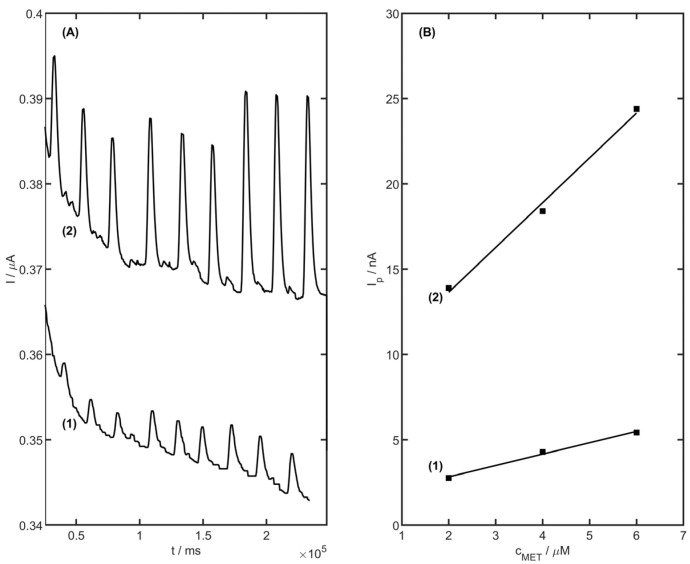
Metoprolol calibration chart (**A**) and curve (**B**) in the concentration range of 2 to 6 µM on unmodified (1) and YSZNd-Cb-Nafion modified GC (2) for the amperometric parameters of measurements under flow injection conditions.

Flow injection analysis with amperometric detection was also used for MET determination. The optimal potential for the working electrode was chosen as 1350 mV, and the optimal flow rate of the acetate buffer (pH 4.0) as equal to 1.5 mL min^−1^. The measurements were recorded on the bare glassy carbon electrode disc (GC) and the GC was modified with an YSZNd-CB-Nafion suspension. The signals of metoprolol determination under flow conditions were registered in the range of 2 to 6 µM of MET concentration. The diagrams were recorded for bare and modified electrodes (Figure 12). The signal for the modified electrode was ca. 10 times higher, compared to the bare electrode. The RSD of performed amperometric measurements was equal to 2.4%. The calibration curve of MET determination on unmodified and YSZNd-CB-Nafion-modified GC electrodes by FIA is presented in Figure 9B. The obtained slope of the regression line for the unmodified electrode and modified with YSZNd-CB-Nafion were 0.67 ± 0.06 nA µM^−1^, intercept 1.49 ± 0.01 µM, r = 0.993, slope 2.62 ± 0.02 nA µM^−1^, intercept 8.41 ± 0.39 µM, and r = 0.994, respectively. The YSZNd-CB-Nafion modified GC was applied for the first time for MET determination under flow analysis conditions. The MET concentration in the pharmaceutical product was determined under flow injection analysis. The measurement was carried out using the calibration method. The calculated recovery was in the range 101–103%, which suggests the yttria-stabilized zirconia doped with neodymium-carbon black-Nafion modified GC electrode for metoprolol determination under flow injection amperometric analysis is possible to use.

## 4. Conclusions

In this work, the voltammetric method for highly sensitive metoprolol determination is reported. For the first time, a new electrochemical sensor based on a GC electrode modified with yttria-stabilized zirconia doped with neodymium, carbon black and Nafion was used. The proposed electrochemical sensor presents several advantages, such as the simplicity of the fabrication process, short preparation time (15 min) of electrodes, cost-effectiveness and reproducibility. The modification enabled an improvement in sensitivity of up to 15 times compared to the carbon black-modified GC electrode. The measurement conditions such as the supporting electrolyte and instrumental parameters, as well as stripping parameters, were optimized. The influence of interferents on the metoprolol determination was also investigated. The LOD and LOQ values were computed and for t_acc_ = 105 s, LOD and LOQ were equal to 2.9 nM and 6.6 nM, respectively. This is the best result obtained, compared to other voltammetric sensors for MET determination that have been described in the literature. The proposed method has been employed for the determination of MET in pharmaceutical formulations, urine and plasma samples. The recoveries obtained were in the range of 96–108%, which suggest the analytical usefulness of the yttria-stabilized zirconia doped with neodymium-carbon black-Nafion modified GCE electrode for metoprolol determination in real samples. Moreover, the proposed sensor was also used in flow injection amperometric analysis. The obtained signal of metoprolol for the YSZNd-CB-Nafion-modified glassy carbon disc electrode was 10 times higher, compared to the unmodified electrode. The recovery in the range of 101–103% for the pharmaceutical formulation suggests the potential to use YSZNd-CB-Nafion-modified GC measuring MET under flow injection conditions. On the whole, the flow injection measurements significantly reduced the time and cost analysis. In summary, the proposed voltammetric sensor, based on an YSZNd nanopowder, carbon black, Nafion-modified glassy carbon electrode, has proven to have a wide range of applicability in the field of pharmaceutical, urine and plasma analysis. Overall, the developed voltammetric method for metoprolol determination may be a useful tool in routine MET measurements, both in stationary and flow conditions.

## Figures and Tables

**Table 1:** The comparison of the capacitance and charge-transfer-resistance parameter values obtained for the modified and unmodified electrode based on EIS measurements.

	GCE	CB/GCE	Nafion/GCE	CB-Nafion/GCE	YSZNd-CB-Nafion/GCE
C, [µF]	0.028	0.673	0.078	2.310	1.008
R_ct_, [kΩ]	1.34	1.08	22.05	2.97	2.06

**Table 2:** Comparison of different developed sensors used in determination of MET.

Electrode	Technique	Electrolytic Solution	Linear Range [µM]	LOD [nM]	Reference
Ga_2_-ZnO/BDDE	DPV	B-R buffer pH 7.0	0.99–38.5	75.3	[15]
TiO_2_/MIP/CPE	CV	Phosphate buffer pH 7.0	10–120	5 µM	[16]
Cathodically pretreated BDD	DPV	Lactate buffer pH 4.0	0.38–22	77	[17]
		B-R buffer pH 7.0	1.2–23	34	
MIP/MWCNTs/PGE	DPV	B-R buffer pH 3.0	0.06–490	2.9	[24]
NAF-CNT-GCE	AdDPV	Phosphate buffer pH 7.0	0.0702–9	51	[25]
GRE/PtNPS/NFN	AdDPV	B-R buffer pH 7.0	0.0144–7.50	4.30	[28]
YSZNd-CB-Nafion GCE	DPV	Acetate buffer pH 4.0	0.01–0.20	2.9	This work

Ga_2_-ZnO/BDDE—glutardialdehyde-zinc oxide modified boron-doped diamond electrode; TiO_2_/MIP/CPE—Titanium dioxide molecularly imprinted polymer modified carbon paste electrode; Cathodically pretreated BDD—cathodically pretreated boron-doped diamond; MIP/MWCNTs/PGE—multi-walled carbon nanotubes molecularly imprinted polymer-modified pencil graphite electrode; NAF-CNT-GCE—Nafion-carbon nanotube-nanocomposite film modified glassy carbon electrode; GRE/PtNPS/NFN—graphene/platinum nanoparticles/nafion nanocomposite modified electrode; YSZNd-CB-Nafion GCE—YSZ doped with Nd nanopowders—carbon black nanoparticles—Nafion-modified glassy carbon electrode.

**Table 3:** Results of metoprolol determination in pharmaceutical product, urine and plasma samples.

Sample	Added [µM]	Found [µM]	Recovery [%]
Tablet	0	0.98 ± 0.04	-
	1	2.04 ± 0.01	106
	2	3.02 ± 0.09	102
	3	4.07 ± 0.16	103
Urine diluted 100×	0	ND	-
	1	0.96 ± 0.05	96
	2	1.96 ± 0.08	98
	3	3.13 ± 0.07	104
Plasma diluted 100×	0	ND	-
	1	1.08 ± 0.03	108
	2	1.94 ± 0.15	98
	3	3.24 ± 0.08	108

## Data Availability

The data presented in this study are available on request from the corresponding author. The data is not publicly available due to an excessive amount of data in the repository.
